# Dissection of Functional Modules of AT-HOOK MOTIF NUCLEAR LOCALIZED PROTEIN 4 in the Development of the Root Xylem

**DOI:** 10.3389/fpls.2021.632078

**Published:** 2021-04-06

**Authors:** Minji Seo, Ji-Young Lee

**Affiliations:** ^1^School of Biological Sciences, College of Natural Science, Seoul National University, Seoul, South Korea; ^2^Plant Genomics and Breeding Institute, Seoul National University, Seoul, South Korea

**Keywords:** AT-HOOK MOTIF NUCLEAR LOCALIZED PROTEIN 4, intercellular movement, protein–protein interaction, xylem, root apical meristem (RAM)

## Abstract

Xylem development in the *Arabidopsis* root apical meristem requires a complex cross talk between plant hormone signaling and transcriptional factors (TFs). The key processes involve fine-tuning between neighboring cells, mediated *via* the intercellular movement of signaling molecules. As an example, we previously reported that AT-HOOK MOTIF NUCLEAR LOCALIZED PROTEIN (AHL) 4 (AHL4), a member of the 29 AT-hook family TFs in *Arabidopsis*, moves into xylem precursors from their neighbors to determine xylem differentiation. As part of the effort to understand the molecular functions of AHL4, we performed domain swapping analyses using AHL1 as a counterpart, finding that AHL4 has three functionally distinctive protein modules. The plant and prokaryotes conserved (PPC) domain of AHL4 acts as a mediator of protein–protein interactions with AHL members. The N-terminus of AHL4 is required for the regulation of xylem development likely *via* its unique DNA-binding activity. The C-terminus of AHL4 confers intercellular mobility. Our characterization of modules in the AHL4 protein will augment our understanding of the complexity of regulation and the evolution of intercellular mobility in AHL4 and its relatives.

## Introduction

**FIGURE 1:**
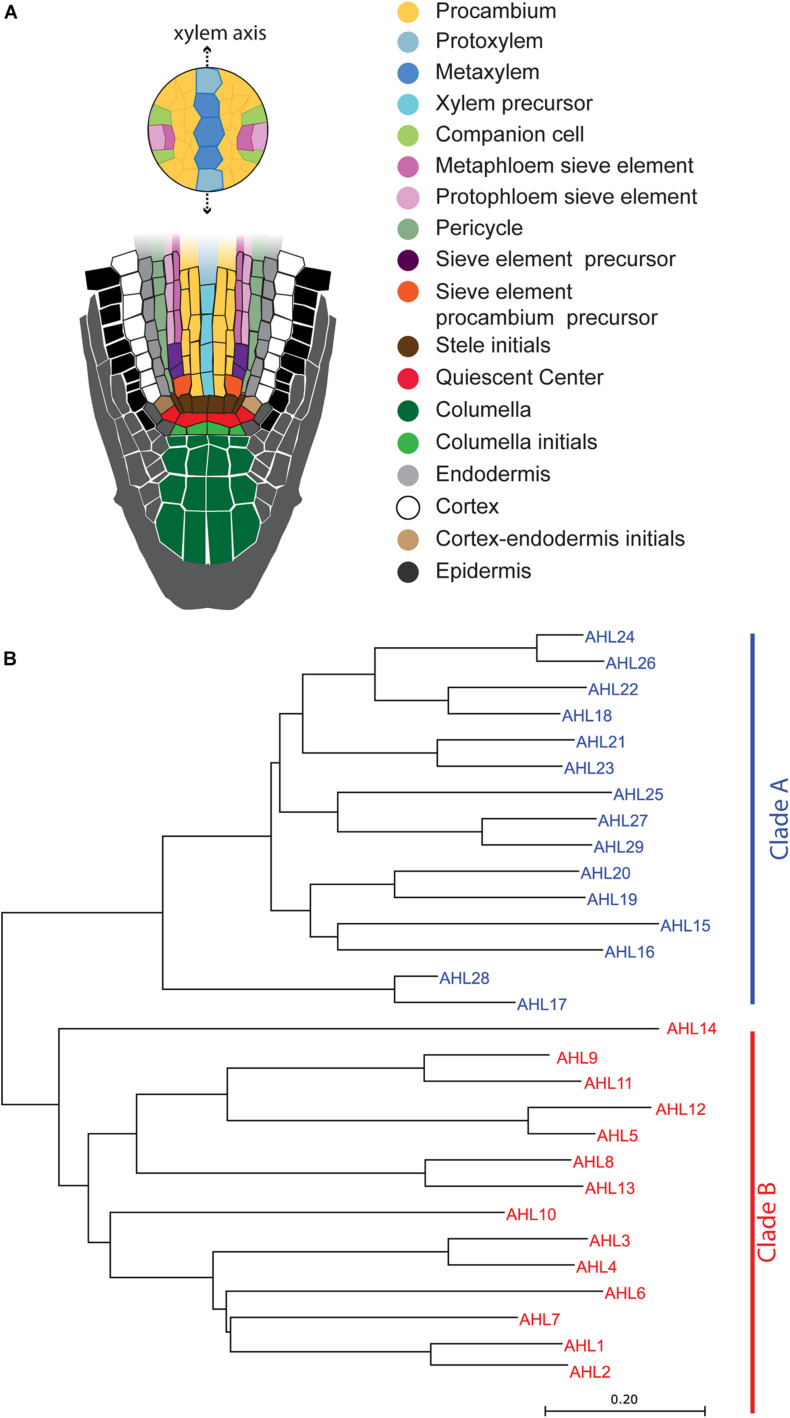
Schematic illustration of the *Arabidopsis* root apical meristem and phylogenetic tree of AT-HOOK MOTIF NUCLEAR LOCALIZED PROTEINS (AHLs): **(A)** schematic illustration of the *Arabidopsis* root apical meristem. **(B)** Phylogenetic tree of AHL proteins in *Arabidopsis*. The evolutionary history was inferred using the neighbor-joining method (Saitou and Nei, 1987). The optimal tree with the sum of the branch length being 10.17264217 is shown. The tree is drawn to scale, with branch lengths in the units identical to those of the evolutionary distances used to infer the phylogenetic tree.

The vascular system plays a key role in the transport and mechanical support processes in vascular plants. It is composed of two major tissues, the xylem and phloem, and undifferentiated stem cells between them. The organization of the vascular system is well defined in the *Arabidopsis* root apical meristem (Figure 1A). It is bisymmetrically organized with xylem vessels running through the center and two phloem poles located perpendicular to the xylem axis. On the xylem axis, protoxylem cells differentiate in the periphery and metaxylem cells differentiate in the center. Procambium cells, undifferentiated stem cells, occupy the region between the xylem and phloem (Mahonen et al., 2000). Five xylem vessels usually differentiate in a single row while neighboring procambium cells remain undifferentiated. This suggests the presence of a tight regulatory process that defines the xylem axis.

Given the importance of the vascular system in the success of plants in terrestrial environments, detailed molecular mechanisms underlying the development of this system have become available in recent years (Vaughan-Hirsch et al., 2018; Chiang and Greb, 2019). Findings pertaining to the *Arabidopsis* root indicate that the processes generating xylem precursors and determining their cell fates and that differentiation require extensive interplay among transcription factors (TFs) and cell–cell signaling (Seo et al., 2020).

Several TFs provide positional information by directly moving between cells (Gallagher et al., 2014; Gundu et al., 2020). Among them, SHORTROOT (SHR) broadly impacts the specification and patterning of root tissues inside the epidermis and root cap (Benfey et al., 1993; Laurenzio et al., 1996; Helariutta et al., 2000; Sabatini et al., 2003; Carlsbecker et al., 2010). *SHR* is expressed in parts of the stele, e.g., the xylem precursors, procambium, and neighboring pericycle cells (Helariutta et al., 2000; Sena et al., 2004). Translated SHR protein moves into adjacent phloem pole and cell layers outside the stele, specifically the quiescent center (QC), cortex–endodermis initial (CEI), and endodermis (Nakajima et al., 2001). In the phloem pole, SHR induces asymmetric cell division for the formation of proto- and meta-phloem sieve elements (Kim et al., 2020). SHR moving outside of the stele maintains the QC, promotes asymmetric cell division in the CEI, specifies endodermis cell fate, and patterns xylem vessels. To regulate these processes, SHR induces the expression of *SCR* and BIRD family genes, including *JACKDAW* (*JKD*), *MAGPIE* (*MGP*), and *NUTCRACKER* (*NUC*) (Levesque et al., 2006; Moreno-Risueno et al., 2015), and then interacts with their proteins.

SHR, SCR, and one of the BIRD members form trimeric protein complexes which play a role in downstream gene expression (Gallagher et al., 2004; Cui et al., 2007; Welch et al., 2007; Long et al., 2015). SHR–SCR with either MGP or NUC promotes asymmetric cell division of the CEI to generate the cortex and endodermis layers (Welch et al., 2007; Long et al., 2015). This requires the activation of D-type cyclin in the CEI (Sozzani et al., 2010). Spatial restriction of D-type cyclin to the CEI is achieved *via* the competitive inhibitory binding of JDK to SHR–SCR (Long et al., 2015). A recent study of the structure of the SHR–SCR complex (Hirano et al., 2017) suggested that the α/β core subdomain of the SHR protein can specifically recognize BIRD proteins. Spatiotemporally coordinated interactions between BIRD proteins and SHR–SCR enable tissue patterning and cell division at the proper time and in the correct places (Moreno-Risueno et al., 2015; Long et al., 2017). Furthermore, this protein–protein interaction is critical for controlling the intercellular movement of SHR (Gallagher et al., 2004; Cui et al., 2007; Long et al., 2015). SHR–SCR–BIRD complexes target the nuclei, which blocks the movement of SHR to an adjacent cell layer.

Some mobile TFs are under the regulation of plant hormones. As the auxin gradient is established during embryogenesis, MONOPTEROS (MP) activates the expression of several downstream targets, including *TARGET OF MONOPTEROS 7* (*TMO7*) (Schlereth et al., 2010). TMO7, a small basic helix-loop-helix protein, moves into the hypophysis and promotes cell division of the hypophysis for QC formation (Lu et al., 2018). In postembryonic *Arabidopsis* roots, mutual inhibitory actions between auxin and cytokinin contribute to the bisymmetric organization of the xylem and phloem (Bishopp et al., 2011). Cytokinin on the phloem side promotes the expression of *PHLOEM EARLY DOF 1* (*PEAR1*) and its homologs. PEARs then move to neighboring procambial cells and there suppress the expression of HD-ZIP IIIs, which function as repressors of cell division (Miyashima et al., 2019). Our group previously reported that two AT-HOOK MOTIF NUCLEAR LOCALIZED PROTEIN (AHL) family members, AHL3 and AHL4, are also possible mobile outputs of cytokinin to regulate the xylem axis in the *Arabidopsis* root (Zhou et al., 2013). During this regulation process, the AHL4 protein interacts with AHL3. Because interaction and movement are frequently discovered as characteristics of mobile TFs involved in cell type patterning, dissecting these two aspects is important to understand the molecular mechanisms.

In *Arabidopsis*, there are 29 genes encoding AT-hook TF family members (Consortium, 2011; Zhao et al., 2014), which are classified into two major clades: clades A and B. Some AHLs in clade A are known to regulate plant growth and development processes, such as hypocotyl and petiole elongation, leaf senescence, and gibberellin synthesis (Matsushita et al., 2007; Street et al., 2008; Xiao et al., 2009; Favero et al., 2020). Based on the amino acid sequence alignment of AHL proteins, all AHL members contain two highly conserved motifs: the AT-hook motif and the plant and prokaryotes conserved (PPC) domain (Fujimoto et al., 2004). The AT-hook motif contains a conserved palindromic core with a sequence of three amino acids (Arg-Gly-Arg) that can bind to the minor groove of the AT-rich B form of DNA (Reeves and Nissen, 1990; Huth et al., 1997). The PPC domain is approximately 120 amino acids in length and is highly conserved among AHL proteins (Fujimoto et al., 2004; Lin et al., 2007). The PPC domain in AHL29 of clade A has been shown to be involved in the protein–protein interactions with other AHL members (Zhao et al., 2013).

To understand how AHL4 in clade B regulates xylem development, we defined the AHL4 full-length protein into three domains and investigated the molecular function of each domain. For the domain analyses, we chose AHL1 as a counterpart, which is relatively close to AHL4 in the phylogenetic tree but does not have intercellular mobility, and generated a series of chimeric proteins between AHL1 and AHL4. Multifaceted analyses of the behaviors of these chimeras enabled us to understand how each domain serves as a functional module of AHL4.

## Materials and Methods

### Plant Material and Growth Condition

*Arabidopsis thaliana* ecotype Columbia-0 (Col-0) was used throughout this research. *ahl4* mutant (SALK_124619) was obtained from the ABRC in a previous study (Zhou et al., 2013). Seedlings for confocal imaging were germinated and grown vertically on the surface of the Murashige and Skoog (MS) solid medium supplemented with 1% sucrose. Before plating on the MS media, the seed surface was sterilized. Seeds on the MS media were stratified for 2 days at 4°C and then grown vertically in a growth chamber that was constantly maintained at 22°C with a cycle of 16-h days and 8-h nights.

### Inference of the Phylogeny of AT-Hook Family Transcription Factors

All AHL family protein sequences used in this report were downloaded from TAIR^1^. The full-length amino acid sequences were subsequently aligned with Clustal Omega^2^ (Madeira et al., 2019). The phylogenetic relationship was analyzed using the MEGA X program (Kumar et al., 2018), with the neighbor-joining method (Saitou and Nei, 1987). The tree is drawn to scale, with branch lengths in the same units as those of the evolutionary distances used to infer the phylogenetic tree. The evolutionary distances were computed using the Poisson correction method (Zuckerkandl and Pauling, 1965) and are expressed here in units of the number of amino acid substitutions per site.

### Cloning of the PPC Domain and AHL Protein Coding Sequences

All the *AHL* protein coding regions used in yeast two-hybrid assays, except for *AHL3* and *AHL4*, were amplified from the root cDNA of Col-0 using polymerase chain reactions (PCR). PPC domains of *AHL3* and *AHL4* coding regions were amplified from *AHL3* and *AHL4* cloned in pENTR221, respectively (Zhou et al., 2013). PCRs were performed using Phusion^®^, High-Fidelity DNA Polymerase (New England Biolabs). Forward primers and reverse primers used in PCRs are indicated in Supplementary Table 3. Amplified DNAs from PCR were purified by HiGene^TM^ Gel and PCR purification system (BIOFACT). Except for *AHL1*, other *AHL* genes were inserted into pENTR^TM^/D-TOPO^TM^ vector by pENTR/D-TOPO Cloning Kit (Invitrogen). The *AHL1* gene was cloned by BP Clonase reaction. The TOPO and BP reactions were proceeded following the manufacturer’s instruction. The reaction mixture was transformed into *Escherichia coli* TOP10-competent cells and clones with expected cDNA inserts were identified. All the constructs were confirmed by Sanger sequencing.

### *AHL1* Promoter Cloning

Gateway technology (Invitrogen) was used for cloning the *AHL1* promoter. Two-step PCRs and the BP Clonase reaction were used to clone the upstream intergenic region of *AHL1* (*pAHL1*) into the pDONR P4_P1R vector. The primary PCR amplified the region encompassing the upstream and downstream sequences of *pAHL1* using the genomic DNA of Col-0 as a template. The primary PCR amplicant was used as a template for the second PCR to amplify *pAHL1* attached to attB sites. Phusion^®^, High-Fidelity DNA Polymerase (New England Biolabs) was used for the PCRs. For primary PCR, promoter AHL1 _3kb_F and promoter AHL1_R primers were used, and for secondary (containing attB site) PCR, promoter AHL1_3kb_Sense and promoter AHL1_Antisense were used. Sequence information about the primers is presented in Supplementary Table 3. The BP cloning reaction and *E. coli* transformation were conducted following the manufacturer’s instructions. The *AHL1* promoter clone was finally confirmed by Sanger sequencing.

### AHL1–AHL4 Chimeric Protein Cloning

For the cloning of chimeric proteins, the Gibson method was used (Gibson et al., 2009; Gibson, 2011). A set of primers were designed by NEBuilder^®^, ^3^. The primers used in the PCRs are indicated in Supplementary Table 3. To clone AHL4-4-1, GB_AHL1_D3_F/GB_AHL1_D3_R (template: AHL1 CDS in p221) were used for insert fragment cloning and GB_p221_AHL4_D1D2_F/GB_p221_AHL4_D1D2_R (template: AHL4 CDS in p221) were used for vector fragment cloning. To clone AHL4-1-1, GB_AHL4 _D1_F/GB_AHL4_D1_R (template: AHL4 CDS in p221) were used for insert fragment cloning and GB_p221_AHL1 _D2D3_F/GB_p221_AHL1_D2D3_R (template: AHL1 CDS in p221) were used for vector fragment cloning. To clone AHL1-1-4, GB_AHL4_D3_F/GB_AHL4_D3_R (template: AHL4 CDS in p221) were used for insert fragment cloning and GB_p221_AHL1_D1D2_F/GB_p221_AHL1_D1D2_R (template: AHL1 CDS in p221) were used for vector fragment cloning. To clone AHL1-4-4, GB_AHL1_D1_F/GB _AHL1_D1_R (template: AHL1 CDS in p221) were used for insert fragment cloning and GB_p221 _AHL4_D2D3_F/GB_p221_AHL4_D2D3_R (template: AHL4 CDS in p221) were used for vector fragment cloning. Then, 4 μl of purified insert, 1 μl of purified vector, and 5 μl 2 × Gibson Assembly Master Mix (New England Biolabs)^®^, were mixed and incubated at 50°C for 1 h. The reaction mixture was subsequently transformed into *E. coli* DH5a (dam^+^ strain), and the cloned plasmids from *E. coli* were purified and screened to select the predicted chimera. All the chimera constructs were confirmed by Sanger sequencing.

### Cloning Transcriptional and Translational GFP Fusion Constructs

To generate transcriptional and translational GFP fusion constructs, Multisite Gateway LR cloning was used. Promoters were cloned into the pDONR P4_P1R vector. CDS for translational fusion without a stop codon was cloned into the pDONR221 vector. Free GFP and erGFP cloned into pDONR P2R_P3 were used (Lee et al., 2006). Then, *pAHL1*:erGFP, *pAHL1*:AHL1-GFP, *pSHR*:AHL1-GFP, *pSHR*:AHL4-GFP, *pSHR*:AHL4-4-1-GFP, *pSHR*:AHL4-1-1-GFP, *pSHR*:AHL1-1-4-GFP, *pSHR*:AHL1-4-4-GFP, *pWOL:AHL1*-GFP, and *pWOL*:AHL4-GFP were constructed into dpGreen-BarT (Lee et al., 2006) by means of an LR Clonase^TM^ II Plus enzyme reaction (Invitrogen). *pAHL4*:erGFP and *pAHL4*:AHL4-GFP transgenic plants were generated in a previous study (Zhou et al., 2013).

### Floral Dipping and Transgenic Selection

All constructs in dpGreen-BarT were transformed into *Agrobacterium tumefaciens* GV3101 with pSOUP and were transformed into either the wild type or *ahl4* by the floral dipping method (Clough and Bent, 1998). Every transgenic line containing a target transgene was selected with a 2,000-fold diluted Basta (Bayer Crop Science) solution on soil or 10 μg/ml of glufosinate ammonium (Fluka) on MS media.

### Yeast Vector Cloning

Each of the AHL3, AHL4, and PPC domains of AHL3 and AHL4 in pENTR221 was cloned into both pDEST22, a prey vector for fusion with the GAL4 activation domain, and pDEST32, a bait vector for fusion with GAL4 DNA-binding domain, using Gateway LR recombination. Other AHLs and AHL1–AHL4 chimeras, AHL4-4-1, AHL4-1-1, AHL1-1-4, and AHL1-4-4, in pENTR221 were cloned into pDEST22. For LR reaction, 3 μl of each donor plasmid, 1 μl of pDEST22 or pDEST32, and 1 μl of LR II clonase were mixed and incubated for 1 h at room temperature. Then, the reaction mixture was transformed into *E. coli* TOP10-competent cells and screened for clones with expected cDNA inserts. All the constructs were confirmed by Sanger sequencing.

### Yeast Two-Hybrid Assay

A ProQuest two-hybrid system (Invitrogen) was used for the yeast two-hybrid analysis. All of the procedures were performed according to the manufacturer’s standard protocol. Recombinant hybrid proteins were tested for self-activation. Plasmids between the pDEST32 and the pDEST22 vector were used as negative control. Plasmid DNA pairs between pEXP32-Krev1 and pEXP22-RalGDS were used as controls for positive interactions. To judge the protein–protein interaction, the 3-amino-1,2,4-triazole (3-AT) assay method was used. For the 3-AT assay, each yeast transformant was placed into 1.5 ml of SD^2–^ (Leu^–^/Trp^–^) liquid media. After incubation for 2 days at 30°C, the OD_600_ value was measured using a biophotometer spectrometer (Eppendorf). Next, every yeast culture was diluted to an OD_600_ value of 0.1 by adding pure SD^2–^ (Leu^–^/Trp^–^) media. These diluted transformants were dropped onto SD^2–^ (Leu^–^/Trp^–^) media, SD^3–^ (Leu^–^/Trp^–^/His^–^) media, SD^3–^ (Leu^–^/Trp^–^/His^–^) media with 10 mM 3-AT, SD^3–^ (Leu^–^/Trp^–^/His^–^) media with 20 mM 3-AT, and SD^3–^ (Leu^–^/Trp^–^/His^–^) media with 40 mM 3-AT. These yeast droplets on the selection media were incubated at 30°C for 2 days.

### Vibratome Sectioning of Roots for Confocal Microscopy

For *Arabidopsis* xylem pattern phenotyping, 5 DAT (days after transfer to growth chamber from stratification) seedlings were used. Five to six seedlings overlaid straight on a MS plate were pulled together and then dipped into 4% low-melting temperature SeaPlaque^®^, Agarose (Lonza), which was melted in 1 × PBS buffer (pH 7.5). Next, the seedlings in the 4% agarose solution were placed in disposable base molds (30 mm × 24 mm × 5 mm). The solidified agarose was cut into a block and sectioned using a vibratome (Leica VT1000S), resulting in thicknesses in the range of 100–120 μm. For observation of the cell boundaries under a confocal microscope, each slice was stained with 10 μg/ml of a Calcofluor white M2R (Sigma-Aldrich) solution.

### Confocal Microscopy

To visualize the GFP protein, 5 DAT seedlings were stained with 10 μg/ml of a propidium iodide (PI) solution (Life Technologies) for 2 min and imaged with a confocal microscope. Subsequently, a 500 × PI solution (5 mg/ml) was prepared and diluted with a 1 × PI solution in water before staining. Images were taken on a Carl Zeiss LSM700 and a Leica TCS SP8 confocal microscope with an argon-ion laser (488 nm excitation and 509 nm emission for GFP; 493 nm excitation and 636 nm emission for PI; 349 nm excitation and 420 nm emission for Calcofluor white M2R).

### Statistical Analysis

All statistical analyses were performed using RStudio v.1.4.1103. A non-parametric chi-square test of goodness of fit was conducted to determine the *p*-value of each dataset. Bar graphs were generated by GraphPad Prism v.8.4.0 (Motulsky and Christopoulos, 2004).

## Results

### The PPC Domain of AHL4 Mediates Protein–Protein Interaction

A total of 29 AHL proteins in *Arabidopsis* can be classified into two major clades, clade A and clade B (Figure 1B), as defined by Zhao et al. (2014). These clades are supported by major differences in amino acid sequences of the PPC domains of the AHLs. Clade A has a type of PPC domain which starts with Leu-Arg-Ser-His, and clade B has another type of PPC domain which starts with Phe-Thr-Pro-His (Zhao et al., 2014). The PPC domain of the AHL proteins in clade A was found to mediate the protein–protein interaction (Zhao et al., 2013). However, the PPC domain sequences between clade A and clade B are quite different; therefore, it remains unknown as to whether the PPC domain of clade B also serves to mediate the interaction between AHL proteins.

**FIGURE 2:**
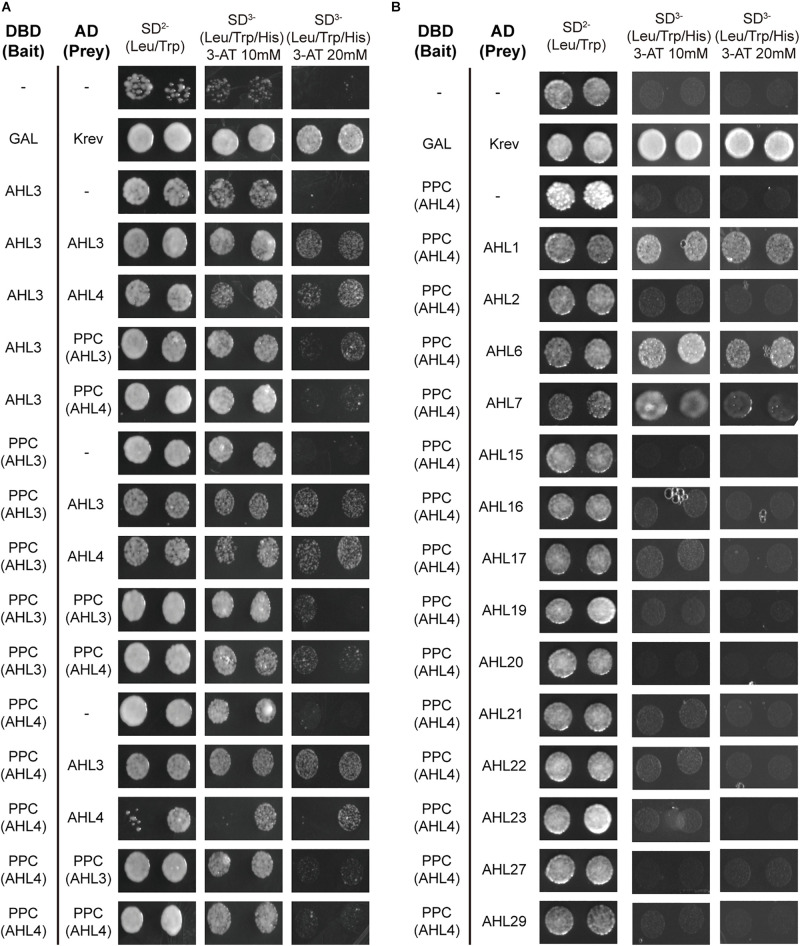
Plant and prokaryotes conserved (PPC) domain of AHL4 mediates protein–protein interaction specific to clade B AHLs. **(A)** Analysis of the PPC domain of AHL3 and AHL4 in the protein–protein interactions using yeast two-hybrid assays. **(B)** Analysis of the PPC domain of AHL4 during interactions with other AHLs using a yeast two-hybrid assay. Left column, a pair of interactors; upper row, series of selection media. DBD (bait), DNA-binding domain; AD (prey), activation domain.

AHL3 and AHL4 are clade B AHLs. To define the role of the PPC domain in the clade B AHLs, we cloned the PPC domains of AHL3 and AHL4 into yeast expression vectors and then analyzed the interactions between the AHL3/4 proteins and the cloned PPC domains. A series of 3-AT was used to prevent autoactivation by the bait. We found that the PPC domains of AHL3 and AHL4 interact well with full-length AHL3/AHL4 proteins (Figure 2A). The criterion of protein–protein interaction was whether a yeast colony appeared on the SD3^–^ media with 20 mM of 3-AT. We extended these assays to other 14 AHL members, finding that the PPC domain of AHL4 does not interact with that of the AHL in clade A, whereas it does interact with AHLs belonging to the same subclade as AHL4, except for AHL2 (Figure 2B). These data suggest that the PPC domain in the AHL4 protein functions as a key mediator of protein–protein interactions to form homomeric or heteromeric proteins and that it provides specificity to interact with AHL proteins in the same clade.

### AHL1 and AHL4 Show Differences in Spatial Expression Patterns and Intercellular Mobility Levels

In the phylogenetic analysis, AHL3 and AHL4 belong to the subclade that includes AHL1, AHL2, AHL6, and AHL7 (Figure 1B). While *AHL3*, *AHL4*, and *AHL6* showed enriched expression levels in the xylem precursor in *Arabidopsis* roots, *AHL1*, *AHL2*, and *AHL7* showed broad expression levels in multiple cell types (Supplementary Figure 1; Zhang et al., 2019). For further analyses of AHL4 protein domains, we selected AHL1 and compared its behavior with that of AHL4.

First, we aimed to define the transcriptional domain of *AHL1* and the intercellular mobility of AHL1 proteins in the root meristem. To this end, we cloned the 3,265-bp-long upstream intergenic region of *AHL1* (*pAHL1*) and attached the endoplasmic reticulum-targeted green fluorescence protein (erGFP). This construct, *pAHL1:erGFP*, which we call transcriptional fusion, was introduced into wild type Col-0. We also made translational fusion lines in Col-0 which express the AHL1 protein fused with a free GFP driven by *pAHL1*. In our confocal microscopy observations, the GFP signal of the *AHL1* transcriptional fusion lines was very low, making it challenging for us to discern the cell layers with GFP expression from those with autofluorescence (Supplementary Figures 2A,B). Nevertheless, the GFP signal was higher in the epidermis and stele region than in the cortex and endodermis (Supplementary Figure 2B). The GFP intensity in the translational fusion lines was much higher than that in the transcriptional fusion lines (Supplementary Figure 2C). Due to the major difference in the GFP intensity levels between the transcriptional and translational fusion lines, we could not determine the intercellular mobility of AHL1. We compared the *AHL1* expression patterns with those in *AHL4* transcriptional (Supplementary Figure 2D) and translational fusion lines (Supplementary Figure 2E). The spatial expression of *AHL4* transcriptional fusion was restricted to the subset of the stele, while AHL4 translational fusion GFP was broadly found in the stele, consistent with a previous report (Zhou et al., 2013).

**FIGURE 3:**
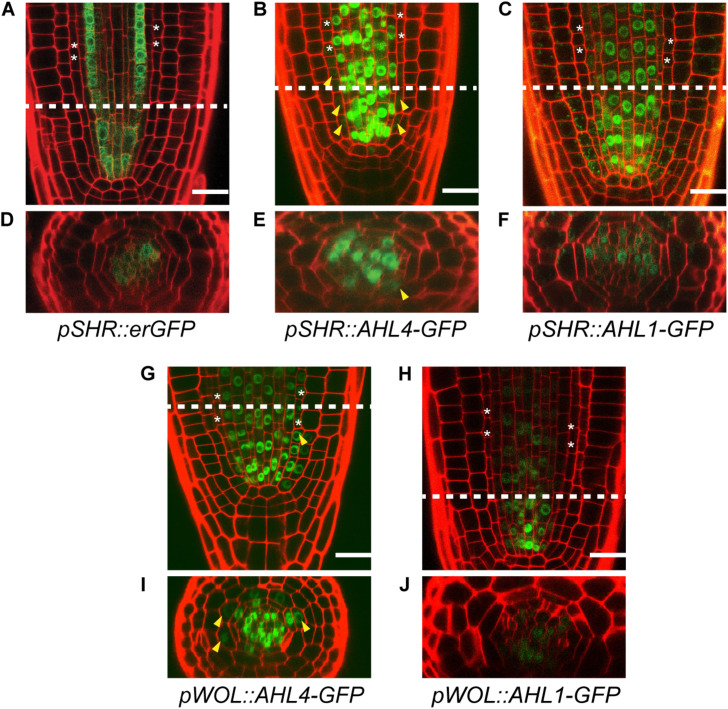
Comparison of the intercellular movements of AHL1 and AHL4 under the *SHR* and *WOL* promoters. Longitudinal views of root apical meristems expressing *pSHR:erGFP* **(A)**, *pSHR:AHL4-GFP* **(B)**, and *pSHR:AHL1-GFP* **(C)**. **(D–F)** Cross-sectional images of the dashed-line positions of panels **(A–C)**. Longitudinal views of root apical meristems expressing *pWOL:AHL4-GFP* **(G)** and *pWOL:AHL1-GFP* **(H)**. **(I,J)** Cross-sectional images on the dashed-line positions of panels **(G,H)**. White asterisks, endodermis; yellow arrowheads, GFP moved to the endodermis; scale bars = 20 μm.

The intercellular mobility of AHL1 was unclear when it was examined with its own promoter. Thus, we employed the promoter of *SHR*, which is well defined. We expressed erGFP under the *SHR* promoter (*pSHR:erGFP* in the Col-0) as a non-mobile control (Figure 3A) (Gallagher et al., 2004) and compared its expression domains with those of GFP translationally fused with AHL1 and AHL4 in each case. The expression levels of these proteins were imaged in five to seven individuals of at least five independent T2 lines. *pSHR:erGFP* started GFP expression broadly right above the QC and then became restricted to the xylem and procambium (Figure 3A). We observed AHL4-GFP throughout the stele and endodermis in all five transgenic lines analyzed (Figure 3B). However, the expression of AHL1-GFP was found only in the stele cells and not in the endodermis (Figure 3C). Z-stack images of each transgenic line were consistent with the longitudinal images (Figures 3D–F). We also noted that the expression level of the AHL1 protein was remarkably lower than that of the AHL4 protein. Nevertheless, our results collectively indicate that AHL1 is not mobile between cells, in contrast to AHL4. To reconfirm this finding, we also checked the expression patterns of GFP fused to either AHL1 or AHL4 under the *WOODEN LEG* (*WOL*) promoter (Figures 3G–J) (Mahonen et al., 2000). Consistent with AHL4/AHL1-GFP driven by the *SHR* promoter, AHL4-GFP expressed under the *WOL* promoter expanded its domain to the endodermis, while AHL1-GFP did not (Figures 3G–J).

### Design of Chimeric Proteins Between AHL4 and AHL1 to Identify Functional Modules

**FIGURE 4:**
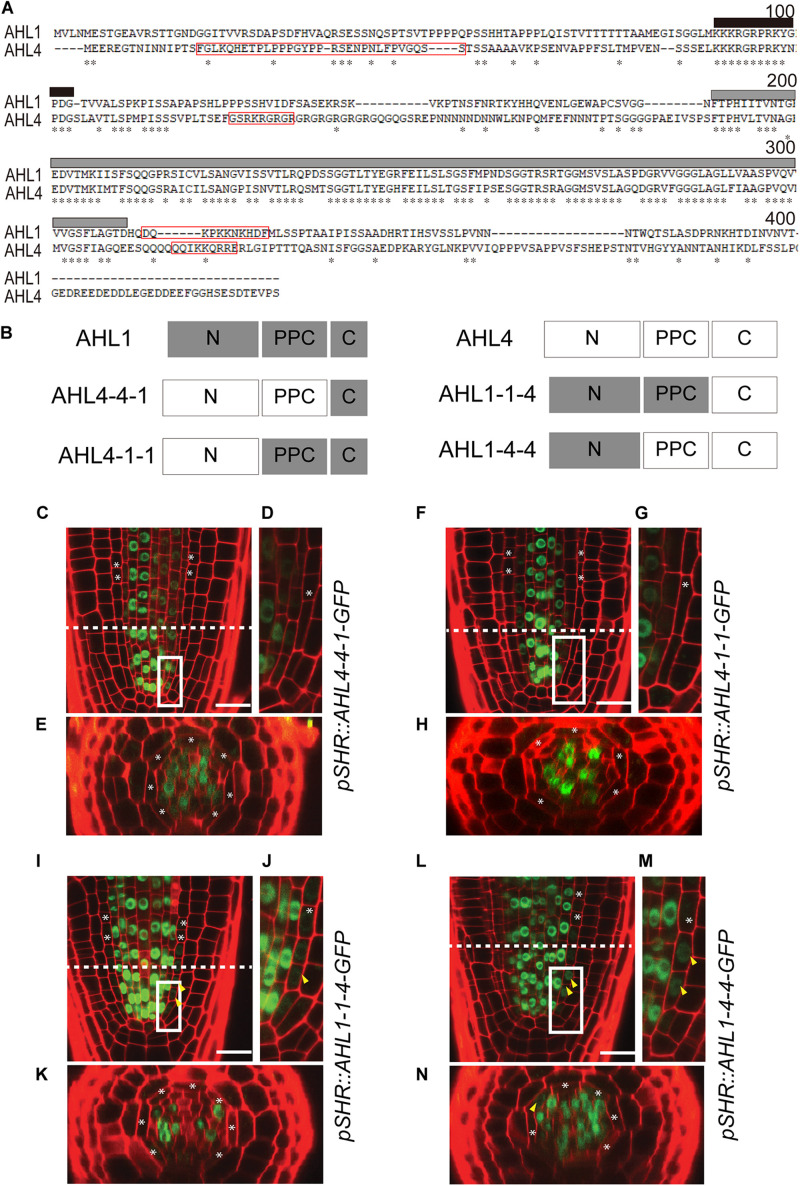
Intercellular movement of AHL1–AHL4 chimeric proteins: **(A)** Amino acid sequence alignment of AHL1 and AHL4 using the AlignX program (Lu and Moriyama, 2004). Asterisks, identical amino acids; red box; NLS sequence; black box, AT-hook motif; gray box, PPC domain. **(B)** Schematic illustration of four chimeric proteins between AHL1 and AHL4. Dark gray box, domain from the AHL1 protein; white box outlined in the light gray, domain from the AHL4 protein. **(C–N)** Confocal microscopy of roots expressing chimeric proteins in the wild type Col-0. **(C–E)** *pSHR:AHL4-4-1-GFP*, **(F–H)** *pSHR:AHL4-1-1-GFP*, **(I–K)** *pSHR:AHL1-1-4-GFP*, and **(L–N)** *pSHR:AHL1-4-4-GFP*. **(D,G,J,M)** Magnified images of regions outlined in white in panels **(C,F,I,L)**. **(E,H,K,N)** Cross-sectional images of the dashed-line positions of panels **(C,F,I,L)**. White asterisks, endodermis; yellow arrowheads, GFP moved into the endodermis; scale bar = 20 μm.

Proteins consist of modules (domains) with distinctive structural/functional features (Lin et al., 2005, 2007). AHL members are defined by a highly conserved PPC domain in the middle and one or two DNA-binding AT-hook domains in the N-terminus. To determine the functional modules of AHL4, its amino acid sequence was compared with AHL1, which does not have intercellular mobility. The amino acid alignment showed three distinctive regions separated by a PPC domain in the middle (Figure 4A). In the alignment, the N-terminus and C-terminus regions separated by the PPC domain are dissimilar between AHL1 and AHL4; however, there is a well-conserved AT-hook motif located in the N-terminus region. In our search for a nuclear localization signal (NLS) in the AHL1 and AHL4 protein sequences using the NLS Mapper (Kosugi et al., 2009), one NLS in the C-terminus of AHL1, two in the N-terminus of AHL4, and one in the C-terminus of AHL4 were detected (Figure 4A). To characterize the functional modules of AHL4, we divided the AHL1 and AHL4 protein sequences into three domains: N-terminus, PPC domain, and C-terminus. Then, we designed four chimeric proteins, each of which had partial sequences from both the AHL1 and AHL4 proteins by means of Gibson cloning (Figure 4B). The first of these, AHL4-4-1, had the AHL4 N-terminus, the AHL4 PPC domain, and the AHL1 C-terminus. The second, AHL4-1-1, had the AHL4 N-terminus, AHL1 PPC domain, and AHL1 C-terminus. The third, AHL1-1-4, had the AHL1 N-terminus, AHL1 PPC domain, and AHL4 C-terminus. The last domain, AHL1-4-4, had the AHL1 N-terminus, AHL4 PPC domain, and AHL4 C-terminus.

It was previously shown that the interaction between AHL4 and AHL3 proteins affects the intercellular movement of the AHL4 protein (Zhou et al., 2013). Accordingly, we examined whether four chimeric proteins still interact with the AHL3 protein as one indication of the maintenance of functional integrity. To do this, we cloned the chimeric proteins and AHL3 into yeast expression vectors and analyzed the interactions between each of the chimeric proteins and AHL3 using a yeast two-hybrid assay (Supplementary Figure 3). It was found that AHL1 and all four chimeric proteins interact with the AHL3 protein. Therefore, creating chimeric proteins did not affect the protein–protein interaction capacity.

### C-Terminus Domain of AHL4 Confers Intercellular Mobility

After confirming that all chimeric proteins from AHL1 and AHL4 interacted with the AHL3 protein (Supplementary Figure 3), we generated transgenic lines expressing each of the chimeric proteins to study their cell-to-cell mobility characteristics. We introduced the following constructs, *pSHR:AHL4-4-1-GFP*, *pSHR:AHL4-1-1-GFP*, *pSHR:AHL1-1-4-GFP*, and *pSHR:AHL1-4-4-GFP*, into the wild type Col-0 background. Then, we observed the localization of GFP proteins in T2 seedling roots of each transgenic line under a confocal microscope. For AHL4-4-1-GFP (Figures 4C–E) and AHL4-1-1-GFP (Figures 4F–H), GFP was restricted to the stele. In contrast, AHL1-1-4-GFP (Figures 4I–K) and AHL1-4-4-GFP (Figures 4L–N) were observed outside of the stele.

### The N-Terminus Domain of AHL4 Is Required for the Regulation of Xylem Development

Next, we investigated whether any of these four chimeric proteins can complement the *ahl4* mutant phenotype. In a previous paper, we reported that the *ahl4* mutant shows a higher frequency of the extra-xylem phenotype than the wild type; however, this report lacked a quantitative analysis (Zhou et al., 2013).

**FIGURE 5:**
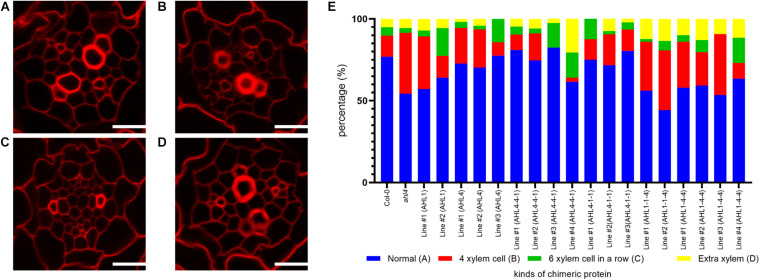
Xylem phenotype recovery by chimeric proteins that are expressed in the stele of the *ahl4* mutant: **(A–D)** Four typical phenotypes of xylem arrangements categorized in the root. Normal phenotype **(A)**, “four xylem cells” phenotype **(B)**, “six xylem cells in a row” phenotype **(C)**, and “extra-xylem” phenotype **(D)**. Scale bars = 20 μm. **(E)** Distribution of the xylem phenotypes of the wild type, *ahl4*, and *ahl4* expressing each chimeric protein, as categorized in **(A–D)**. *n* = 39∼85. All of the detailed scoring data are presented in Supplementary Table 1.

To analyze the xylem phenotype in a quantitative manner, we cross-sectioned the root differentiation zone of wild type *Arabidopsis* seedlings using a vibratome, stained the sections with Calcofluor white, and then imaged them under a confocal microscope (Figures 5A–D). Based on this quantitative phenotyping, we categorized xylem organizations into four types. We considered large cells with thickened cell walls as differentiated xylem vessels. The first type is defined as “normal” because it is the most abundant phenotype in the wild type with two protoxylem cells on both ends of the xylem axis and three metaxylem cells in the center (Figure 5A). The second type is defined as “4 xylem cells,” having only four xylem cells on the xylem axis even after the xylem cell wall thickening process (Figure 5B). The third phenotype is “6 xylem cells in a row,” having an extra xylem cell along the xylem axis (Figure 5C). The last phenotype is called “extra-xylem,” having a differentiated extra protoxylem or metaxylem cell present outside the single row of xylem cells (Figure 5D). To ensure that the aforementioned types of xylem organization in the root differentiation zone are consistent with the organizations of the xylem precursors in the root meristem, we analyzed the GFP expression levels of typical molecular marker lines in the root meristem (Supplementary Figure 4). These are *pTMO5:erGFP* to denote the xylem axis (Lee et al., 2006), *pARR5:erGFP* for procambium cells (Lee et al., 2006), and *pAHP6:erGFP* for protoxylem cell and two neighboring pericycle cells (Mahonen et al., 2006). This molecular marker analysis suggested that the xylem phenotype can be divided into four classes, consistent with our classification of four xylem cell phenotypes based on cell wall thickening in the differentiation zone.

In our analyses of 39 wild type Col-0 individuals, 77% showed the “normal” xylem type and 23% showed variant xylem types. When we analyzed 66 individuals of the *ahl4* mutant, we found a reduction of the “normal” type to 54% and an increase of variant types. Next, we analyzed *ahl4* introduced with chimeric proteins expressed under the *SHR* promoter. Because the *SHR* promoter drives transcription in the xylem precursor, procambium, and neighboring pericycle, it can cover the region into which the AHL4 protein moves to function. We thus used the same constructs used for the analysis of intercellular mobility to analyze the complementation of the *ahl4* phenotype.

Before checking whether chimeric proteins can rescue *ahl4* or not, we analyzed the cases of *pSHR:AHL4-GFP; ahl4* and *pSHR:AHL1-GFP; ahl4.* In the AHL4 case, we noted the recovery of the xylem phenotype to “normal” in three independent transgenic lines. The percentage of “normal” increases from 54 to 73% on average. On the other hand, in the AHL1 case, we noted that there is no meaningful change in the ratio of the xylem phenotype (two individual lines, 54 and 60%) (Figure 5E). Because AHL1 cannot recover the *ahl4* mutant phenotype, we conclude that the molecular function of AHL1 differs from that of AHL4.

In the analysis of transgenic plants with four different chimeric proteins, we determined the rescue of the xylem phenotype based on whether the frequencies of the “normal” phenotype are recovered to those of the wild type and the *pSHR:AHL4-GFP; ahl4* transgenic lines, which are between 73 and 77% (Figure 5E). We also performed chi-square test-based goodness-of-fit analyses between distributions of xylem types of transgenic lines and the reference genotypes (Supplementary Table 1). Under this criterion, we found that the recovery of the xylem phenotype was to “normal” in three out of four independent transgenic lines expressing *pSHR:AHL4-4-1-GFP; ahl4* (two lines with the xylem distribution similar to the wild type; *p* value of goodness-of-fit test > 0.5) and all three lines expressing *pSHR:AHL4-1-1-GFP; ahl4*. In contrast, two independent transgenic lines with *pSHR:AHL1-1-4-GFP; ahl4* (50% of the normal phenotype on average; *p*-values of goodness-of-fit test < 0.5) and four independent transgenic lines with *pSHR:AHL1-4-4-GFP; ahl4* (59% of the normal phenotype on average; *p*-values of goodness-of-fit test < 0.5) did not show a rescue of *ahl4* xylem phenotype. These data indicate that the chimeric proteins containing the AHL4 N-terminus can complement the *ahl4* xylem phenotype. In this context, the N-terminus of AHL4 is important for the specific functions of AHL4 during the xylem development process.

### Intercellular Movement of AHL4 to the Xylem Axis Is Required for Its Regulation of Xylem Development

**FIGURE 6:**
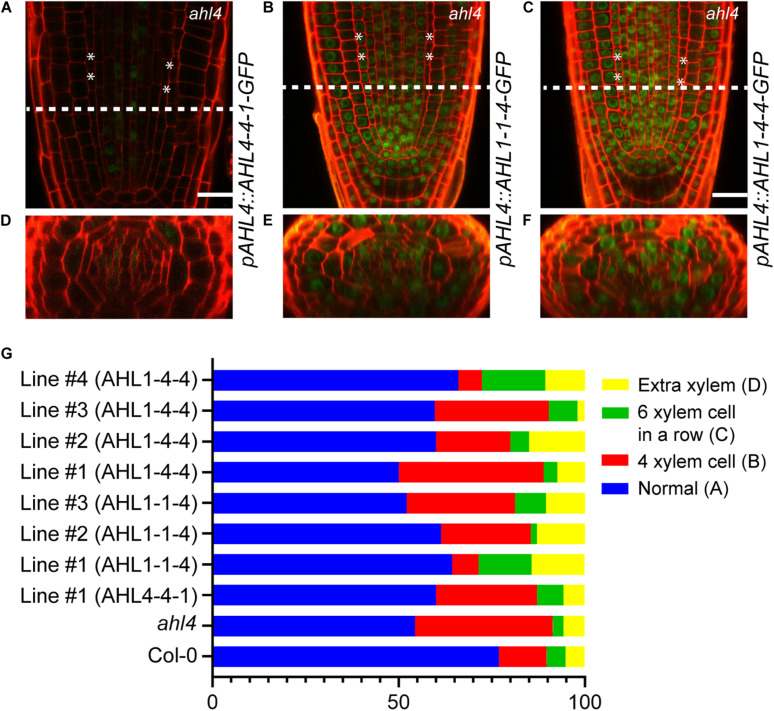
Xylem phenotype rescue of the *ahl4* mutant by chimeric proteins expressed under the *AHL4* promoter. Longitudinal views of root apical meristems expressing *pAHL4:AHL4-4-1-GFP* **(A)**, *pAHL4:AHL1-1-4-GFP* **(B)**, and *pAHL4:AHL1-4-4-GFP* **(C)**. **(D–F)** Cross-sectional images of the dashed-line positions of panels **(A–C)**. Scale bars = 20 μm. **(G)** Distribution of xylem phenotypes of the wild type, *ahl4*, and *ahl4* expressing *pAHL4:AHL4-4-1-GFP*, *pAHL4:AHL1-1-4-GFP*, and *pAHL4:AHL1-4-4-GFP*. Xylem phenotype categorization is identical to that in Figures 5A–D. *n* = 27∼70. All detailed scoring data are presented in Supplementary Table 2.

To reconfirm the importance of the N-terminus of AHL4 in the xylem development process, we analyzed the GFP expression levels and the xylem phenotype of the *ahl4* mutant introduced with *AHL4-4-1-GFP*, *AHL1-1-4-GFP*, or *AHL1-4-4-GFP* under the *AHL4* promoter. Given that the C-terminus of AHL4 confers intercellular mobility (Figure 4), we expected that the immobile AHL4-4-1-GFP protein would only be in the procambium area, while GFP fused to AHL1-1-4 or AHL1-4-4 would be mobile and would be found broadly in the stele. Consistent with our prediction, confocal microscopy indicated that AHL4-4-1-GFP was in the stele but excluded from the xylem axis (Figures 6A,D), while AHL1-1-4-GFP and AHL1-4-4-GFP were found throughout the stele (Figures 6B,C,E,F). The intercellular mobility of AHL1-1-4 and AHL1-4-4 appeared to be more extensive than that of AHL4 because the GFP fusion of the former two expanded not only throughout the stele but also to the ground tissue, epidermis, quiescent center, and root cap.

Subsequently, we analyzed whether those chimeric proteins could recover the xylem phenotype in *ahl4*. Based on complementation analyses of chimeric proteins expressed under the *SHR* promoter (Figure 5E), AHL4-4-1 expressed under the *AHL4* promoter was predicted not to recover the *ahl4* phenotype because it cannot move into xylem precursor cells even though it has a functional domain in the N-terminus. AHL1-1-4 and AHL1-4-4 were also predicted not to rescue the *ahl4* phenotype because these two proteins do not have functional domains in the N-terminus even though they move into the xylem precursors. As predicted, one independent line expressing *pAHL4:AHL4-4-1-GFP; ahl4* showed only 60% of the “normal” phenotype. Three independent lines expressing *pAHL4:AHL1-1-4-GFP; ahl4* showed only 59% of the “normal” phenotype. Likewise, four independent lines expressing *pAHL4:AHL1-4-4-GFP* showed 59% of the “normal” phenotype (Figure 6G and Supplementary Table 2).

## Discussion

In this study, we characterized the functional domains of AHL4, one of the 29 AHLs in *Arabidopsis*. AHLs are largely classified into two clades, clades A and B, based on the amino acid sequences of the PPC domain. The PPC domain in clade A has been characterized as a mediator of the protein–protein interactions between AHL members in clade A (Zhao et al., 2013). In the N-terminus region outside the PPC domain, there are one or two AT-hook motifs, a condition required for DNA binding (Fujimoto et al., 2004). Other than the AT-hook motif, the N-terminus and C-terminus regions outside of the PPC domain are highly variable among AHLs.

AHL4 belongs to clade B and has one AT-hook motif. Our group reported that AHL4 controls xylem development in the root meristem by moving from the procambium to the xylem precursor (Zhou et al., 2013). However, how the functions of AHL4 are differentiated from those of other AHLs remains elusive. To address this, we divided the AHL4 protein into three domains and investigated the function of each domain. First, we isolated the PPC domains of AHL3 and AHL4 and examined their interactions with AHL3 and AHL4 as well as other AHLs (Figure 2). Our yeast two-hybrid assays suggest that the PPC domains alone can interact with the AHL3 or AHL4 protein (Figure 2A). Thus, like the PPC domain in clade A, the PPC domain in clade B appears to mediate the protein–protein interactions among AHLs. Moreover, we found that the PPC domain of AHL4 does not interact with the AHLs in clade A, whereas it does interact with AHL1, AHL6, and AHL7 in clade B (Figure 2B). This finding indicates that the PPC domain of AHL4 confers the specificity of interactions exclusively with the AHLs in clade B. Interestingly, the PPC domain of AHL4 does not interact with the AHL2 protein despite that AHL2 belongs to clade B.

Next, we constructed chimeric proteins while employing AHL1, which is closely related to AHL4 in the phylogeny but does not have intercellular mobility. Analyses of the chimeric proteins between AHL1 and AHL4 provided important clues related to the definitions of the functions of each domain in AHL4. Visual inspections of *pSHR:AHL4-4-1-GFP* and *pSHR:AHL4-1-1-GFP* indicated that these two types of chimeric proteins do not move from the stele to the endodermis (Figures 4C–H). In contrast, our analysis of *pSHR:AHL1-1-4-GFP* and *pSHR:AHL1-4-4-GFP* indicated the movement of GFP-fused proteins from the stele to endodermis cells (Figures 4I–N). Moreover, a visual inspection supported our contention that the movement of GFP fused to AHL1-4-4 is more pronounced than that of AHL1-1-4 (Figures 4I–N). These data indicate that the AHL4 C-terminus domain is responsible for the intercellular mobility of AHL4. Because GFP with the AHL4 C-terminus exhibited different frequencies of movement depending on the origin of the attached PPC domain, the PPC domain appears to be capable of influencing the efficiency of intercellular movements. A previous study reported that NLS in the C-terminus and a hydrophobic end part of the PPC domain are required for the nuclear localization of AHL1 (Fujimoto et al., 2004). AHL4 also possesses predicted NLS in the C-terminus domain (Figure 4A), and all the chimeric proteins we examined, including AHL1-1-4, are nuclear localized. In that context, NLS in the C-terminus domain of AHL4 also seems to play a key role for nuclear localization.

This leads to the question of how the development of the xylem is regulated by AHL4. The N-terminus domain of AHL4 contains an AT-hook domain, which is known to be involved in DNA-binding activity (Reeves and Nissen, 1990; Huth et al., 1997). The AHL1 protein expressed under the *SHR* promoter could not rescue *ahl4*’s xylem phenotype. However, when the chimeric protein had the AHL4 N-terminus and others derived from AHL1, it could complement the *ahl4* phenotype if the protein was expressed in the xylem precursors in the root meristem (*pSHR:AHL4-1-1-GFP*; Figure 5E). This complementation did not occur when the chimeric protein could not move into the xylem precursors (*pAHL4:AHL4-4-1-GFP*; Figure 6). These findings collectively indicate that the N-terminus of AHL4 contains AHL4-specific DNA-binding domains and that this DNA binding likely occurs in xylem precursors.

To consolidate these findings further, we expressed GFP-tagged AHL1-1-4 and AHL1-4-4 under the *AHL4* promoter in the *ahl4* mutant background. Consistent with the proposal that the C-terminus of AHL4 confers intercellular mobility, their expression levels expanded to outside of the stele region as well as to the xylem precursors. The degree of domain expansion of these chimeric proteins appeared to be more extensive than that of the intact AHL4 protein. Considering that both chimeric proteins interact with AHL3, as does AHL4, this phenomenon is unlikely due to the enhanced protein mobility caused by the lack of protein–protein interaction. Despite the presence of AHL1-1-4 and AHL1-4-4 proteins in xylem precursors (Figures 6B,C,E,F), these two chimeras failed to complement the *ahl4* xylem phenotype (Figure 6G), highlighting the importance of the AHL4 N-terminus for the AHL4-specific regulation of xylem development.

AHL4 can interact with AHL3 and likely other AHLs in the same clades *via* the PPC domain. Thus, AHL4 may regulate xylem development as a protein complex with other AHLs. Based on a structural analysis of the PPC domain, AHLs are predicted to form heterotrimers (Fujimoto et al., 2004). In such a case, deciphering the structure and components of an AHL complex would be important to understand how it functions. Furthermore, the interaction between the AHL complex and a non-AHL protein appears to be crucial for downstream regulation. For example, AHL22 binds to and recruits a subset of histone deacetylase (HDAC) enzymes to regulate flowering times (Yun et al., 2012). AHL27 and AHL29 interact with TCP4 and TCP13 to regulate hypocotyl elongation (Zhao et al., 2013). In the clade B case, it has been reported that AHL10 directly interacts with highly ABA-induced1 (HAI1), a protein phosphatase that functions in response to drought stress (Wong et al., 2019).

## Conclusion

We found that the molecular functions of AHL4 for xylem development, protein–protein interaction, and intercellular mobility are achieved *via* its N-terminus, middle PPC domain, and C-terminus. These findings indicate that AHL4 (and possibly others, too) is composed of modules, each of which has its unique function. Whether and how such a modular composition of AHL4 and related AT-hook members contribute to their evolution as positional signals for xylem development, and diversification in vascular plants (Zhao et al., 2014), would be interesting topics for further studies.

## Summary Statement

This article reports how the modular organization of the AHL4 protein, an AT-hook family transcription factor in *Arabidopsis*, contributes to its function as an intercellular signal during the root xylem development process.

## Data Availability Statement

The original contributions presented in the study are publicly available. This data can be found here: sequence data from this article can be found in the GenBank/EMBL data libraries under the following accession numbers: AHL1 (AT4G12080), AHL2 (AT4G22770), AHL3 (AT4G25320), AHL4 (AT5G51590), AHL6 (AT5G62260), AHL7 (AT4G00200), AHL15 (AT3G55560), AHL16 (AT2G42940), AHL17 (AT5G49700), AHL19 (AT3G04570), AHL20 (AT4G14465), AHL21 (AT2G35270), AHL22 (AT2G45430), AHL23 (AT4G17800), AHL27 (AT1G20900), and AHL29 (AT1G76500).

## Author Contributions

J-YL and MS: conceptualization, validation, investigation, resources, writing—original draft, and writing—review and editing. MS: methodology, software, formal analysis, and visualization. J-YL: supervision, project administration, and funding acquisition. Both authors: contributed to the article and approved the submitted version.

## Conflict of Interest

The authors declare that the research was conducted in the absence of any commercial or financial relationships that could be construed as a potential conflict of interest.

## Footnotes

1 https://www.arabidopsis.org

2 https://www.ebi.ac.uk/Tools/msa/clustalo

3 http://nebuilder.neb.com

## Supplementary Material

Supplementary Figure 1 Expression patterns of AHL1, AHL2, AHL3, AHL4, AHL6, and AHL7 in representative cell types of Arabidopsis roots. Relative expression patterns of AHL1, AHL2, AHL3, AHL4, AHL6, and AHL7 in the cell-type-specific expression data. Expression values were row-normalized to visualize relative expression patterns along cell types.

Supplementary Figure 2 Comparison of expression domains and intercellular movements of AHL1 and AHL4 in the root apical meristem. **(A–E)** Transcriptional and translational GFP expressions of *AHL1* and *AHL4*. **(A)** Wild type non-transgenic root, **(B)** pAHL1:erGFP, **(C)** *p*AHL1:AHL1-GFP, **(D)** *p*AHL4:erGFP, and **(E)** *p*AHL4:AHL4-GFP. Scale bar 20μm.

Supplementary Figure 3 Interaction between AHL3 and four types of AHL1-AHL4 chimeric proteins. The result of a 3-AT assay of the interaction between *AHL3* and AHL1-4 chimeric proteins is shown. Left column, a pair of interactors; upper row, a series of selection media. DBD (bait), DNA binding domain; AD (prey), Activation domain.

Supplementary Figure 4 Cell-type-specific molecular marker expression levels in four types of xylem organization: **(A–C)** Expressions of TMO5 **(A)**, ARR5 **(B)**, and AHP6 **(C)** of the ‘normal’ type. **(D–F)** Expressions of TMO5 **(D)**, ARR5 **(E)**, and AHP6 **(F)** of the ‘four xylem cell’ type. **(G–I)** Expressions of TMO5 **(G)**, ARR5 **(H)**, and AHP6 **(I)** of the ‘six xylem cell in a row’ type. **(J–L)** Expressions of TMO5 **(J)**, ARR5 **(K)**, and AHP6 **(L)** of the ‘extra-xylem’ type. Xylem phenotype categorization is identical to that in Figures 5A–D. Yellow arrowhead, xylem axis.

Supplementary Table 1 Xylem phenotype scoring of ahl4 introduced with four chimeric proteins under the SHR promoter and statistical analyse.

Supplementary Table 2 Xylem phenotype scoring of ahl4 introduced with four chimeric proteins under the AHL4 promoter and statistical analyses.

Supplementary Table 3 List of primers used in this study.
