# Supplementary figures and images for: Dissection of Functional Modules of AT-HOOK MOTIF NUCLEAR LOCALIZED PROTEIN 4 in the Development of the Root Xylem
Source: Front Plant Sci. 2021 Apr 6;12:632078. doi: 10.3389/fpls.2021.632078 (PMC8056045; doi:10.3389/fpls.2021.632078)

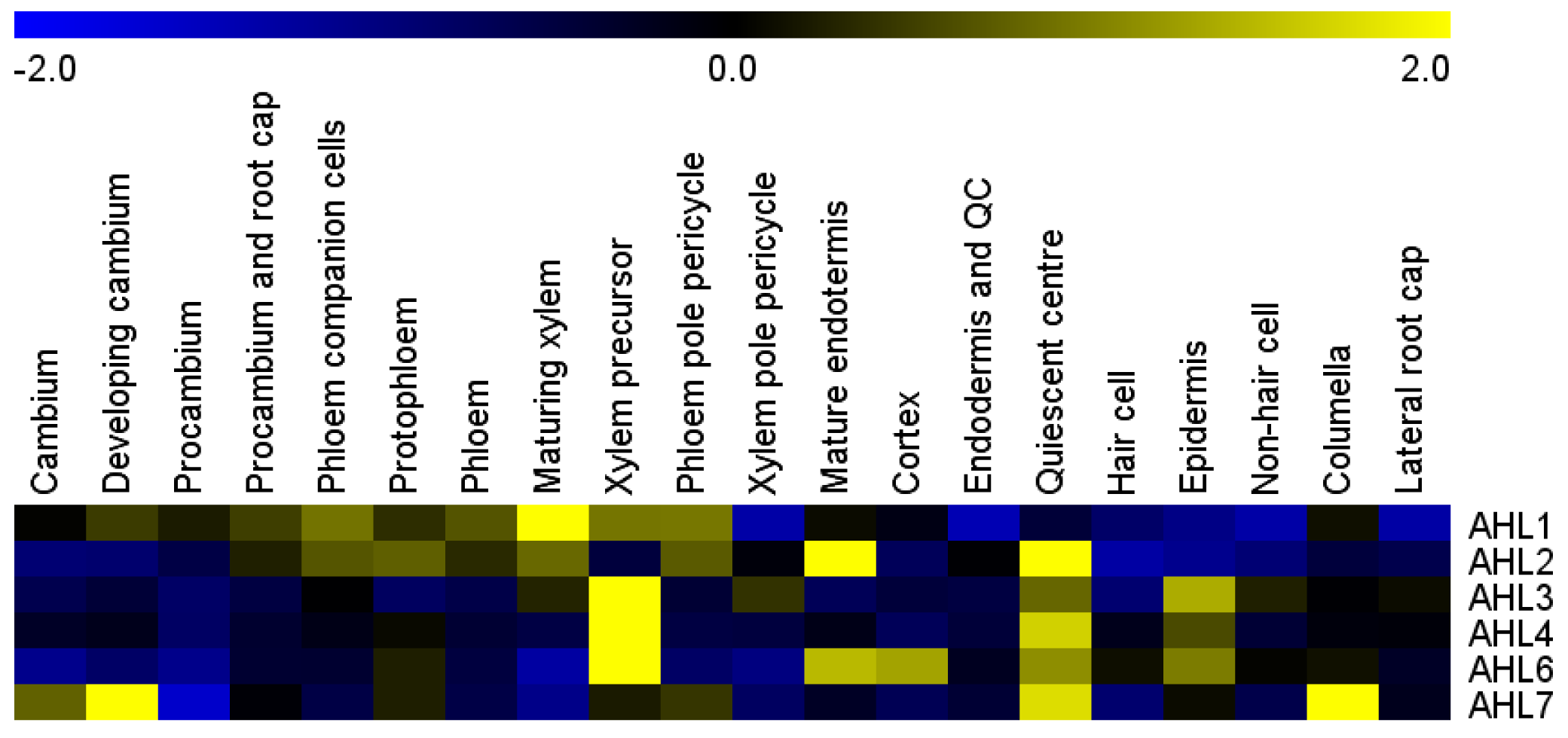

Supplement: Supplementary Figure 1 — Expression patterns of AHL1, AHL2, AHL3, AHL4, AHL6, and AHL7 in representative cell types of Arabidopsis roots. Relative expression patterns of AHL1, AHL2, AHL3, AHL4, AHL6, and AHL7 in the cell-type-specific expression data. Expression values were row-normalized to visualize relative expression patterns along cell types. [file Image_1.TIF]

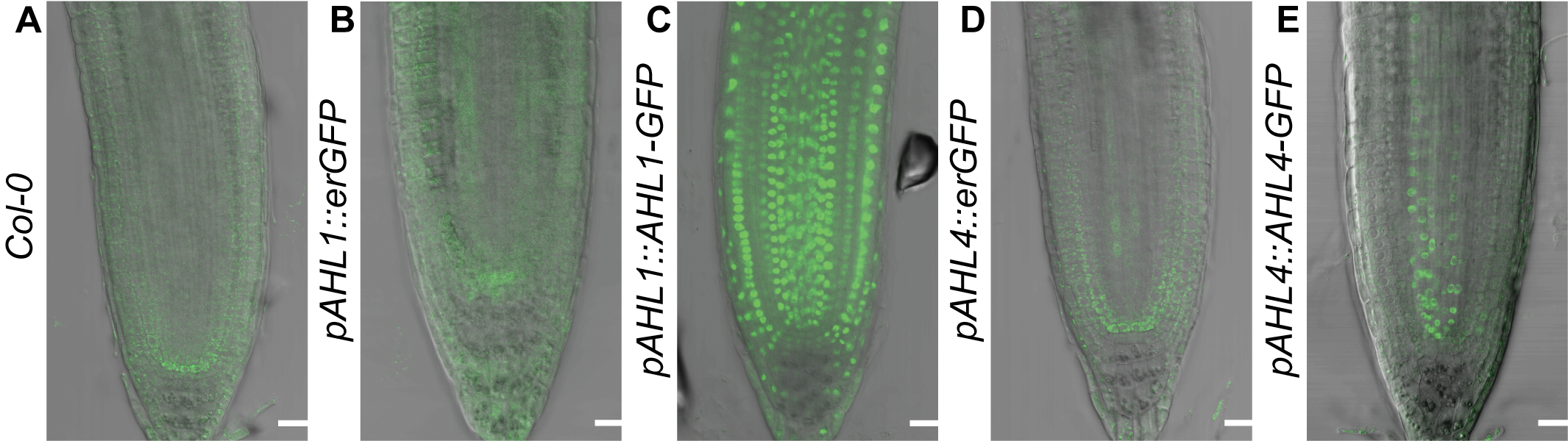

Supplement: Supplementary Figure 2 — Comparison of expression domains and intercellular movements of AHL1 and AHL4 in the root apical meristem. (A–E) Transcriptional and translational GFP expressions of AHL1 and AHL4. (A) Wild type non-transgenic root, (B) pAHL1:erGFP, (C) pAHL1:AHL1-GFP, (D) pAHL4:erGFP, and (E) pAHL4:AHL4-GFP. Scale bar 20μm. [file Image_2.TIF]

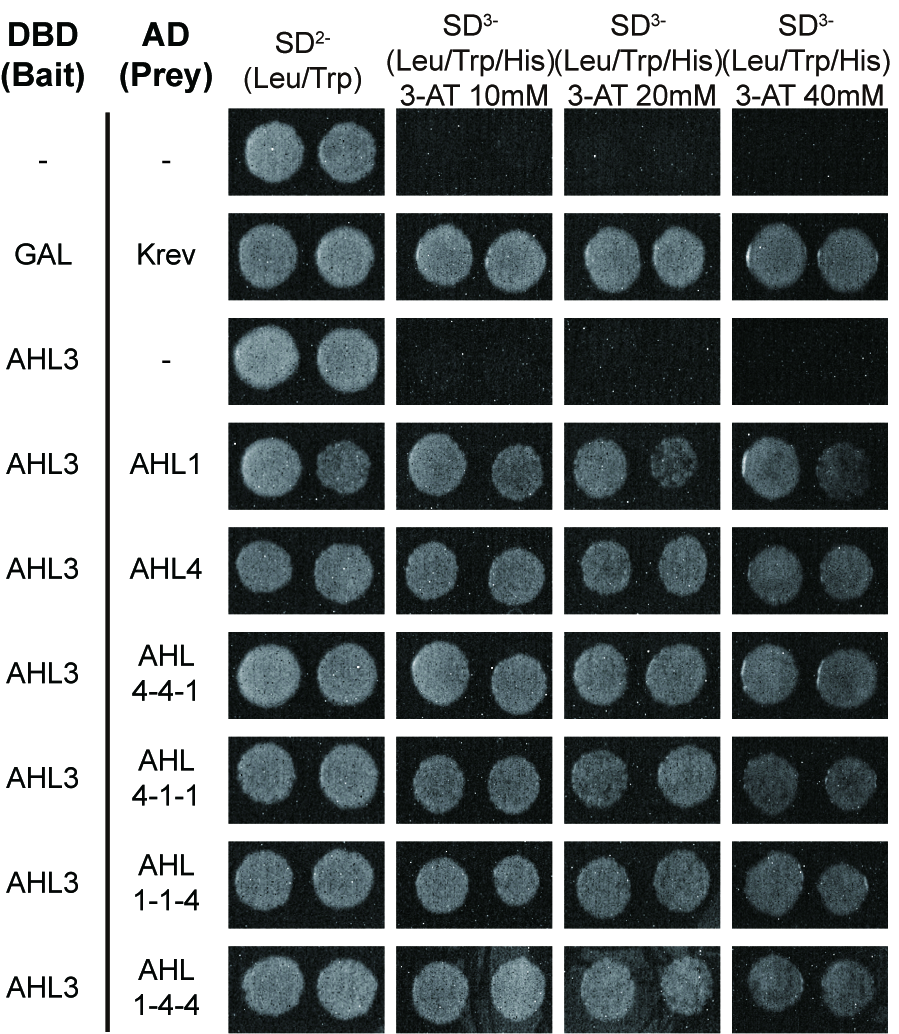

Supplement: Supplementary Figure 3 — Interaction between AHL3 and four types of AHL1-AHL4 chimeric proteins. The result of a 3-AT assay of the interaction between AHL3 and AHL1-4 chimeric proteins is shown. Left column, a pair of interactors; upper row, a series of selection media. DBD (bait), DNA binding domain; AD (prey), Activation domain. [file Image_3.TIF]

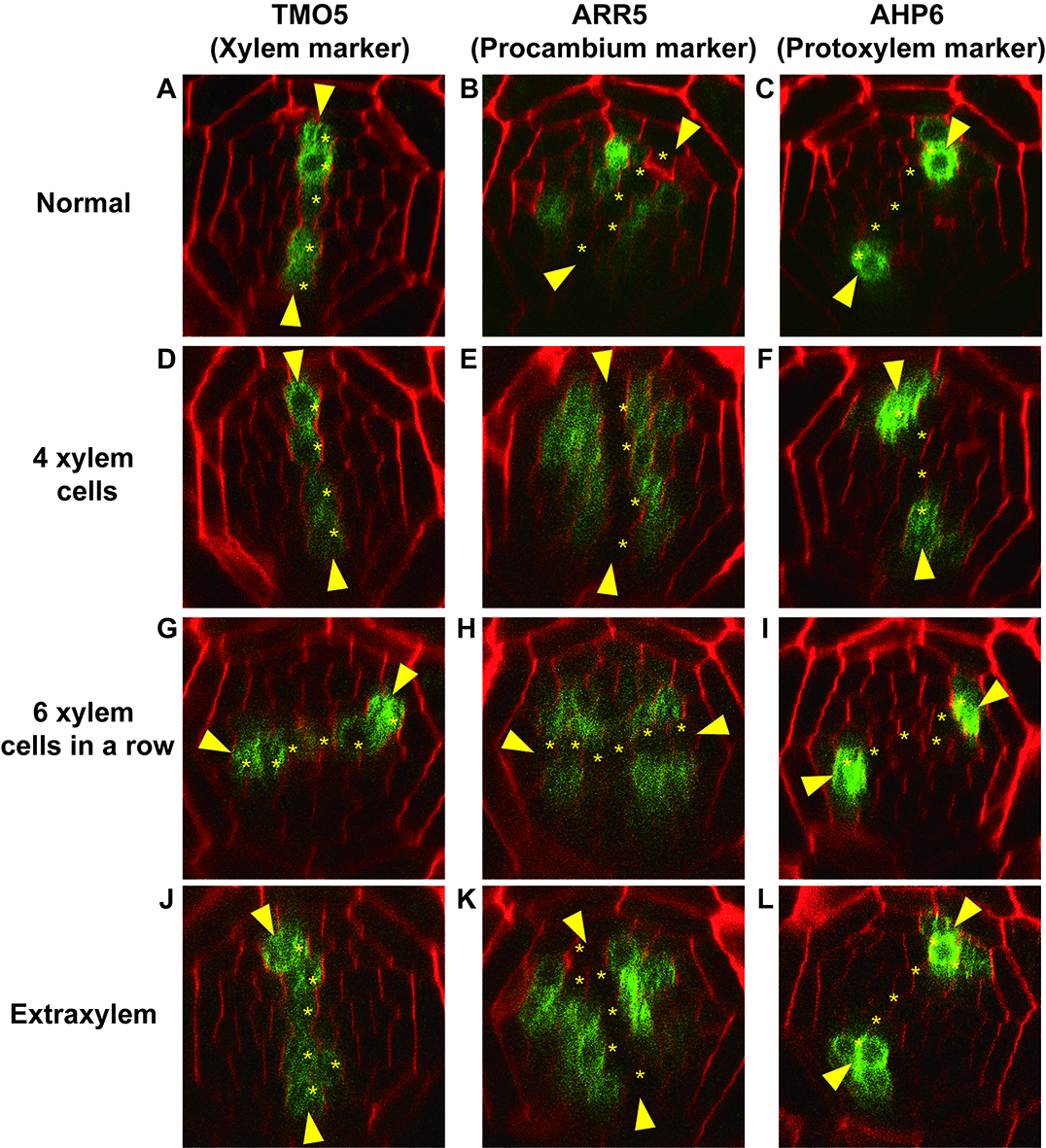

Supplement: Supplementary Figure 4 — Cell-type-specific molecular marker expression levels in four types of xylem organization: (A–C) Expressions of TMO5 (A), ARR5 (B), and AHP6 (C) of the ‘normal’ type. (D–F) Expressions of TMO5 (D), ARR5 (E), and AHP6 (F) of the ‘four xylem cell’ type. (G–I) Expressions of TMO5 (G), ARR5 (H), and AHP6 (I) of the ‘six xylem cell in a row’ type. (J–L) Expressions of TMO5 (J), ARR5 (K), and AHP6 (L) of the ‘extra-xylem’ type. Xylem phenotype categorization is identical to that in Figures 5A–D. Yellow arrowhead, xylem axis. [file Image_4.TIF]
