# Supplementary material for: Dissection of Functional Modules of AT-HOOK MOTIF NUCLEAR LOCALIZED PROTEIN 4 in the Development of the Root Xylem
Source: Front Plant Sci. 2021 Apr 6;12:632078. doi: 10.3389/fpls.2021.632078 (PMC8056045; doi:10.3389/fpls.2021.632078)
Supplement: Supplementary Table 1 — Xylem phenotype scoring of ahl4 introduced with four chimeric proteins under the SHR promoter and statistical analyse. [file Table_1.DOCX]

**Table S1.** Xylem phenotype scoring of *ahl4* introduced with four chimeric proteins under *SHR* promoter and statistical analyses.

|  | Normal | Extra xylem | 6 xylem cell in a row | 4 xylem cell | Total Number | Statistical P value*  (comparison with Col-0 wild type) | Statistical P value*  (comparison with *ahl4* mutant) |
| --- | --- | --- | --- | --- | --- | --- | --- |
| Col-0 | 30 | 2 | 2 | 5 | **39** | 1 | 0.01396 |
| *ahl4* | 19 | 2 | 1 | 13 | **35** | 0.0002926 | 1 |
| *pSHR*::AHL1-GFP in *ahl4* line #1 | 48 | 6 | 3 | 27 | **84** | 1.523x10^-6^ | 0.767 |
| *pSHR*::AHL1-GFP in *ahl4* line #2 | 34 | 3 | 9 | 7 | **53** | 0.001319 | 5.321 x10^-10^ |
| *pSHR*::AHL4-GFP in *ahl4* line #1 | 40 | 1 | 2 | 12 | **55** | 0.1709 | 0.03671 |
| *pSHR*::AHL4-GFP in *ahl4* line #2 | 33 | 2 | 1 | 11 | **47** | 0.1526 | 0.1833 |
| *pSHR*::AHL4-GFP in *ahl4* line #3 | 38 | 0 | 7 | 4 | **49** | 0.009943 | 6.033 x10^-9^ |
| *pSHR*::AHL4-4-1-GFP in *ahl4* line #1 | 34 | 2 | 2 | 4 | **42** | 0.9262 | 0.002065 |
| *pSHR*::AHL4-4-1-GFP in *ahl4* line #2 | 50 | 4 | 2 | 11 | **67** | 0.702 | 0.004935 |
| *pSHR*::AHL4-4-1-GFP in *ahl4* line #3 | 33 | 1 | 6 | 0 | **40** | 0.003793 | 3.842 x10^-9^ |
| *pSHR*::AHL4-4-1-GFP in *ahl4* line #4 | 24 | 8 | 6 | 1 | **39** | 1.137 x10^-6^ | 1.125 x10^-10^ |
| *pSHR*::AHL4-1-1-GFP in *ahl4* line #1 | 36 | 0 | 6 | 6 | **48** | 0.05566 | 1.376 x10^-6^ |
| *pSHR*::AHL4-1-1-GFP in *ahl4* line #2 | 38 | 4 | 1 | 10 | **53** | 0.3352 | 0.04183 |
| *pSHR*::AHL4-1-1-GFP in *ahl4* line #3 | 37 | 1 | 2 | 6 | **46** | 0.8223 | 0.002461 |
| *pSHR*::AHL1-1-4-GFP in *ahl4* line #1 | 32 | 7 | 1 | 17 | **57** | 4.025 x10^-5^ | 0.1446 |
| *pSHR*::AHL1-1-4-GFP in *ahl4* line #2 | 23 | 7 | 3 | 19 | **52** | 4.33 x10^-8^ | 0.04645 |
| *pSHR*::AHL1-4-4-GFP in *ahl4* line #1 | 29 | 5 | 2 | 14 | **50** | 0.003263 | 0.3782 |
| *pSHR*::AHL1-4-4-GFP in *ahl4* line #2 | 32 | 7 | 4 | 11 | **54** | 0.008879 | 0.004195 |
| *pSHR*::AHL1-4-4-GFP in *ahl4* line #3 | 23 | 4 | 0 | 16 | **43** | 6.851 x10^-6^ | 0.5315 |
| *pSHR*::AHL1-4-4-GFP in *ahl4* line #4 | 33 | 6 | 8 | 5 | **52** | 0.000906 | 2.384 x10^-9^ |

* The statistical analysis was performed using nonparametric chi-squared test goodness of fit test of each sample against the wild-type control (Col-0) and *ahl4* mutant. All analyses were done using R program. 1.4.1103. Red color = p value <0.05.
