# Supplementary material for: Dissection of Functional Modules of AT-HOOK MOTIF NUCLEAR LOCALIZED PROTEIN 4 in the Development of the Root Xylem
Source: Front Plant Sci. 2021 Apr 6;12:632078. doi: 10.3389/fpls.2021.632078 (PMC8056045; doi:10.3389/fpls.2021.632078)
Supplement: Supplementary Table 2 — Xylem phenotype scoring of ahl4 introduced with four chimeric proteins under the AHL4 promoter and statistical analyses. [file Table_2.DOCX]

**Table S2.** Xylem phenotype scoring of *ahl4* introduced with four chimeric proteins under *AHL4* promoter and statistical analyses.

|  | Normal | Extra xylem | 6 xylem cell in a row | 4 xylem cell | Total Number | Statistical P value*  (comparison with Col-0 wild type) | Statistical P value*  (comparison with *ahl4* mutant) |
| --- | --- | --- | --- | --- | --- | --- | --- |
| Col-0 | 30 | 2 | 2 | 5 | **39** | 1 | 0.01396 |
| *ahl4* | 19 | 2 | 1 | 13 | **35** | 0.0002926 | 1 |
| *pAHL4::*AHL4-4-1*-GFP in ahl4* line *#1* | 42 | 7 | 5 | 19 | **73** | 0.0007942 | 0.03575 |
| *pAHL4*::AHL1-1-4-GFP in *ahl4* line #1 | 45 | 10 | 10 | 5 | **70** | 9.057 x10^-6^ | 8.497 x10^-13^ |
| *pAHL4*::AHL1-1-4-GFP in *ahl4* line #2 | 38 | 8 | 1 | 15 | **62** | 0.0006976 | 0.02553 |
| *pAHL4*:AHL1-1-4-GFP in *ahl4* line #3 | 25 | 5 | 4 | 14 | **48** | 0.0005756 | 0.05123 |
| *pAHL4*:AHL1-4-4-GFP in *ahl4* line #1 | 27 | 4 | 2 | 21 | **54** | 1.575 x10^-7^ | 0.8888 |
| *pAHL4*::AHL1-4-4-GFP in *ahl4* line #2 | 36 | 9 | 3 | 12 | **60** | 0.001108 | 0.001712 |
| *pAHL4*:AHL1-4-4-GFP in *ahl4* line #3 | 31 | 1 | 4 | 16 | **52** | 0.0007769 | 0.09354 |
| *pAHL4*:AHL1-4-4-GFP in *ahl4* line #4 | 31 | 5 | 8 | 3 | **47** | 0.0004398 | 1.98 x10^-10^ |

* The statistical analysis was performed using nonparametric chi-squared test goodness of fit test of each sample against the wild-type control (Col-0) and *ahl4* mutant. All analyses were done using R program. 1.4.1103. Red color = p value <0.05.
