# Supplementary material for: Dissection of Functional Modules of AT-HOOK MOTIF NUCLEAR LOCALIZED PROTEIN 4 in the Development of the Root Xylem
Source: Front Plant Sci. 2021 Apr 6;12:632078. doi: 10.3389/fpls.2021.632078 (PMC8056045; doi:10.3389/fpls.2021.632078)
Supplement: Supplementary Table 3 — List of primers used in this study. [file Table_3.DOCX]

| Table S3. The list of primers used in this study. | |  |
| --- | --- | --- |
| Oligo Name | Oligo Sequences | Purpose |
| Promoter AHL1 _3kb_F | 5’- ATG CCT AGG CCT ATT AAT TAT GTT TTT TTA-3’ | Promoter AHL1 cloning for 1^st^ PCR |
| Promoter AHL1 _R | 5’-CTC AAC TTG GTG ATG ATA CTT TGT TCT GTT A-3’ | Promoter AHL1 cloning for 1^st^ PCR |
| Promoter AHL1 _3kb_Sense | 5’-GGG GAC AAC TTT GTA TAG AAA AGT TGC TAT GCC TAG GCC TAT TAA T-3’ | Promoter AHL1 cloning for 2^nd^ PCR |
| Promoter AHL1 _Antisense | 5’-GGG GAC TGC TTT TTT GTA CAA ACT TGC GGA ATA CTT GGT GGG TGC-3’ | Promoter AHL1 cloning for 2^nd^ PCR |
| GB_p221_AHL4_D1D2_F | 5’-CAC CCA GCT TTC TTG TAC AAA G-3’ | AHL4-4-1 chimeric gene cloning |
| GB_p221_AHL4_D1D2_R | 5’-CTC CTG ACC CGC TAT AAA AC-3’ | AHL4-4-1 chimeric gene cloning |
| GB_AHL1_D3_F | 5’-ATA GCG GGT CAG GAG GGC ACT GAC CAT CAA GAT CA-3’ | AHL4-4-1 chimeric gene cloning |
| GB_AHL1_D3_R | 5’-ACA AGA AAG CTG GGT GAG TTA CAT TGA CAT TAA TAT CGG TAT G-3’ | AHL4-4-1 chimeric gene cloning |
| GB_p221_AHL1_D2D3_F | 5’-TTC ACA CCT CAT ATA ATC ACA GTC-3’ | AHL4-1-1 chimeric gene cloning |
| GB_p221_AHL1_D2D3_R | 5’-AAA GCC TGC TTT TTT GTA CAA AG-3’ | AHL4-1-1 chimeric gene cloning |
| GB_AHL4_D1_F | 5’-TAC AAA AAA GCA GGC TTT ATG GAG GAG AGA GAA GGA AC-3’ | AHL4-1-1 chimeric gene cloning |
| GB_AHL4_D1_R | 5’-GAT TAT ATG AGG TGT GAA ACT TGG ACT GAC AAT TTC AG-3’ | AHL4-1-1 chimeric gene cloning |
| GB_p221_AHL1_D1D2_F | 5’-TGA TAC CCA GCT TTC TTG TAC-3’ | AHL1-1-4 chimeric gene cloning |
| GB_p221_AHL1_D1D2_R | 5’-CGC TAA AAA ACT TCC TAC AAC-3’ | AHL1-1-4 chimeric gene cloning |
| GB_AHL4_D3_F | 5’-GTA GGA AGT TTT TTA GCG GAA TCG CAG CAG CAG CAG CA-3’ | AHL1-1-4 chimeric gene cloning |
| GB_AHL4_D3_R | 5’-AAG AAA GCT GGG TAT CAG CTT GGA ACC TCG GTG TCA GAT TC-3’ | AHL1-1-4 chimeric gene cloning |
| GB_p221_AHL4_D2D3_F | 5’-TTT ACA CCT CAT GTG CTC AC-3’ | AHL1-4-4 chimeric gene cloning |
| GB_p221_AHL4_D2D3_R | 5’-TGG AGC CTG CTT TTT TGT AC-3’ | AHL1-4-4 chimeric gene cloning |
| GB_AHL1_D1_F | 5’-ACA AAA AAG CAG GCT CCA ATG GTC TTA AAT ATG GAG TCT ACC G-3’ | AHL1-4-4 chimeric gene cloning |
| GB_AHL1_D1_R | 5’-AGC ACA TGA GGT GTA AAA TTA CCA CCG ACG GAG CAA G-3’ | AHL1-4-4 chimeric gene cloning |
| AHL1_F | 5’-GGG GAC AAG TTT GTA CAA AAA AGC AGG CTT TAT GGT CTT AAA TAT GGA-3’ | AHL1 CDS cloning |
| AHL_1_R_NS | 5’-GGG GAC CAC TTT GTA CAA GAA AGC TGG GTA AGT TAC ATT GAC ATT AA-3’ | AHL1 CDS cloning |
| AHL2_F | 5'-CAC CAT GGA GAC TAC CGG AGA AGT TG-3' | AHL2 CDS cloning |
| AHL2_R_NS | 5'-CGT CAA AGT GAT ATT AAA GTC ATG A-3' | AHL2 CDS cloning |
| AHL6_F | 5’-CAC CAT GGA GGA GAA AGG TGA AAT-3’ | AHL6 CDS cloning |
| AHL6_R_NS | 5'-ACC ACT ATG AGA TTG GCT CCT A-3' | AHL6 CDS cloning |
| AHL7_F | 5’-CAC CAT GGA AAC AAG CGA CAG AAT–3’ | AHL7 CDS cloning |
| AHL7_R_NS | 5'-GTC GAC TGG TAA AGA TAT GTT G-3' | AHL7 CDS cloning |
| AHL15_F | 5'-CAC CAT GGC GAA TCC TTG GTG GGT A-3' | AHL15 CDS cloning |
| AHL15_R_NS | 5'-ATA CGA AGG AGG AGC ACG AGG C-3' | AHL15 CDS cloning |
| AHL16_F | 5'-CAC CAT GGC TGG AGG TAC AGC TCT AA-3' | AHL16 CDS cloning |
| AHL16_R_NS | 5'-AGG TTT CGA CAT GAC ACG CTG C-3' | AHL16 CDS cloning |
| AHL17_F | 5'-CAC CAT GAA AGG TGA ATA CAG AGA GC-3' | AHL17 CDS cloning |
| AHL17_R_NS | 5'-GTA TGG CGG TGG AGC TCT GGC-3' | AHL17 CDS cloning |
| AHL19_F | 5'-CAC CAT GGC GAA TCC ATG GTG GAC AG-3' | AHL19 CDS cloning |
| AHL19_R_NS | 5'-AAA TCC TGA CCT AGC TTG AGC CCA A-3' | AHL19 CDS cloning |
| AHL20_F | 5'-CAC CAT GGC AAA CCC TTG GTG GAC GA-3' | AHL20 CDS cloning |
| AHL20_R_NS | 5'-GTA AGG TGG TCT TGC GTG GAC-3' | AHL20 CDS cloning |
| AHL21_F | 5'-CAC CAT GGC TGG TCT CGA TCT AGG CA-3' | AHL21 CDS cloning |
| AHL21_R_NS | 5'-AAA CGG AGC CCT ACC GGC GC-3' | AHL21 CDS cloning |
| AHL22_F | 5'-CAC CAT GGA TCA GGT CTC TCG CTC TC-3' | AHL22 CDS cloning |
| AHL22_R_NS | 5'-GAA AGA TGG TCT CGG AGT TCC C-3' | AHL22 CDS cloning |
| AHL23_F | 5'-CAC CAT GGC TGG TCT TGA TCT AGG CA-3' | AHL23 CDS cloning |
| AHL23_R_NS | 5'-GAA AGG ACC TCT TCC ACC GGA A-3' | AHL23 CDS cloning |
| AHL27_F | 5'-CAC CAT GGA AGG CGG TTA CGA GCA A-3' | AHL27 CDS cloning |
| AHL27_R_NS | 5'-AAA AGG TGG TCT TGA AGG TGT TCC A-3' | AHL27 CDS cloning |
| AHL29_F | 5'-CAC CAT GGA CGG TGG TTA CGA TCA ATC-3' | AHL29 CDS cloning |
| AHL29_R_NS | 5'-AAA GGC TGG TCT TGG TGG TGC G-3' | AHL29 CDS cloning |
| PPC of AHL3_F_CACC | 5’-CCC GGG CCA GAT GGT CGT GTC TTT GGT GGA GGA C-3’ | PPC domain of AHL3 cloning |
| PPC of AHL3_R | 5’-CTC TTG ACC AGC TAT AAA AGT CCC TA-3’ | PPC domain of AHL3 cloning |
| PPC of AHL4_F_CACC | 5’-CAC CAT GTT TAC ACC TCA TGT G-3’ | PPC domain of AHL4 cloning |
| PPC of AHL4_R | 5’-CTC CTG ACC CGC TAT AAA-3’ | PPC domain of AHL4 cloning |
